# Pyrazolo[5,1-*c*][1,2,4]triazole: A Promising Emerging Biologically Active Scaffold in Medicinal Chemistry

**DOI:** 10.3390/ijms26178190

**Published:** 2025-08-23

**Authors:** Beniamin-Nicolae Pintea, Vasilica-Georgiana Panțîr, Valentin Badea, Francisc Péter

**Affiliations:** Department of Applied Chemistry and Engineering of Organic and Natural Compounds, Faculty of Chemical Engineering, Biotechnology, and Environmental Protection, University Politehnica Timișoara, Vasile Parvan Blv. 6, 300223 Timișoara, Romania; bnpintea@hotmail.com (B.-N.P.); georgiana.pantir93@gmail.com (V.-G.P.); francisc.peter@upt.ro (F.P.)

**Keywords:** pyrazolo[5,1-*c*][1,2,4]triazoles, biological activity, toxicity

## Abstract

Nitrogen-containing heterocycles are essential compounds in nature, and their structural and functional diversity inspired the synthesis of a wide range of derivatives with diverse applications as pharmaceuticals, agrochemicals, dyes, polymers, cosmetics, etc. Among them, *N*-fused heterocycles represent an important category, due to their high potential as biologically active agents. Pyrazolo[5,1-*c*][1,2,4]triazoles, a class of nitrogen heterobicycles, have multiple applications as dyes and pigments. Also, a number of compounds containing this structure have been investigated for their biological activities. All the main experimental results published in the literature (both articles and patents) regarding the latter are summarized in this review.

## 1. Introduction

Most pharmaceuticals currently in use are small organic molecules, and, among them, nitrogen-containing heterocyclic scaffolds have shown very useful biological activities against various diseases [1]. Moreover, *N*-heterocyclic structures are widely distributed in nature, including in amino acids, peptides and proteins; nucleotides, nucleosides and nucleic acids; vitamins and many secondary metabolites [2]. Almost one-third of the best-selling therapeutics contain fused heterocyclic structures, justifying the high scientific interest and research effort towards these compounds. *N*-Fused heterocyclic compounds are basic components of several common pharmaceuticals, agrochemicals, plastics, and dyes [3,4].

**Figure 1:**
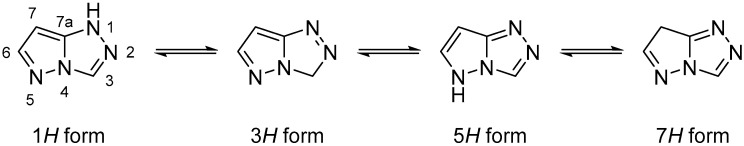
The tautomeric forms of pyrazolo[5,1-*c*][1,2,4]triazole.

Pyrazolo[5,1-*c*][1,2,4]triazoles are a class of N-containing biheterocyclic compounds mainly known for their use in dyes and pigments in a wide variety of fields. The pyrazolo[5,1-*c*][1,2,4]triazole system can theoretically exist in four tautomeric forms, namely 1*H*, 3*H*, 5*H,* and 7*H* (Figure 1); the most widely encountered is the 1*H* form, while the 5*H* form has been reported only in a few compounds, some of which are actually 5-substituted. The remaining two, not fully aromatic forms 3*H* and 7*H*, have been seen almost exclusively in 3,3- and 7,7-disubstituted compounds, respectively.

The numerous methods reported in the literature for the synthesis of the pyrazolo[5,1-*c*][1,2,4]triazole core have been recently reviewed [5,6].

The pyrazolo[5,1-*c*][1,2,4]triazole system combines two frequently used biologically active scaffolds, those of pyrazole and 1,2,4-triazole. Also, as a 5-5 bicyclic system, it is similar in size to other 5-5, 5-6, and 6-6 scaffolds. Not surprisingly, then, a number of biological activity studies have included pyrazolo[5,1-*c*][1,2,4]triazole derivatives, including several annulated analogs. The aim of the present review is to report all the relevant findings of these studies, in order to highlight the biological potential of this scaffold. Principally, the Reaxys and SciFinder databases were used for the literature search, covering the period between 1988 and 2025, targeting structures containing the pyrazolo[5,1-*c*][1,2,4]triazole core and the biological activity of these compounds.

**Note**: This present work is organized based on the biological activity the compounds were tested for, as described below:

1. Acetylcholinesterase inhibition activity
2. Anti-inflammatory activity
3. Analgesic activity
4. Antidiabetic activity
5. Antibacterial activity
6. Antifungal activity
7. Antiviral activity
8. Antiprotozoal activity
9. Anticancer activity
10. C3a receptor binding activity
11. Miscellaneous biological activities
12. Cytotoxicity
13. Ulcerogenic activity

## 2. Acetylcholinesterase Inhibition Activity

**Figure 2:**
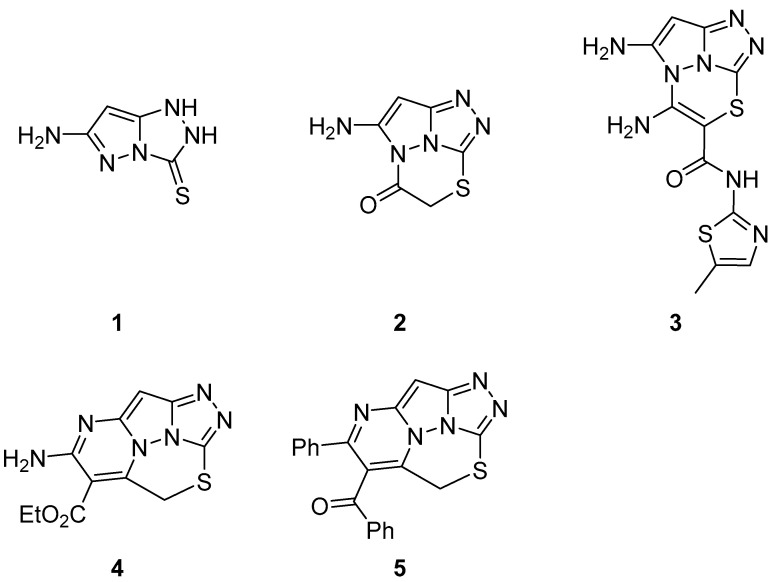
Chemical structures of compounds **1**–**5**.

Compounds **1**–**5** (Figure 2), 6-amino-1,2-dihydro-3*H*-pyrazolo[5,1-*c*][1,2,4]triazole-3-thione **1**, 4-amino-1-thia-3a,6,7,7b-tetraazacyclopenta[*cd*]inden-3(2*H*)-one **2**, 1,7-diamino-*N*-(5-methyl-1,3-thiazol-2-yl)-3-thia-4,5,7a,7b-tetraazacyclopenta[*cd*]indene-2-carboxamide **3**, ethyl 4-amino-2*H*-1-thia-5,7,8,8b,8c-pentaazacyclopenta[*bc*]acenaphthylene-3-carboxylate **4** and phenyl(4-phenyl-2*H*-1-thia-5,7,8,8b,8c-pentaazacyclopenta[*bc*]acenaphthylen-3-yl)methanone **5**, were assayed for acetylcholinesterase inhibition activity [7]. Donepezil was used as reference drug. The results are displayed in Table 1. Compound **1** showed strong acetylcholinesterase inhibition activity, compounds **4** and **5** were moderate acetylcholinesterase inhibitors, while compounds **2** and **3** were weak inhibitors. (Note: This paper is of poor quality; therefore, the information in it should be taken cautiously.)

## 3. Anti-Inflammatory Activity

**Figure 3:**
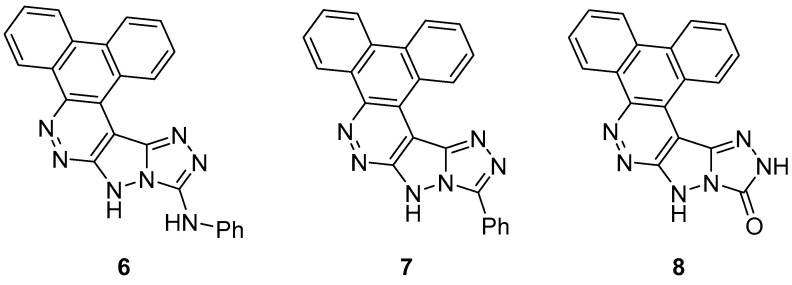
Chemical structures of compounds **6**–**8**.

[1,2,4]Triazolo[4′,3′:1,5]pyrazolo[3,4-c]dibenzo[f,*h*]cinnolines **6**–**8** (Figure 3), *N*-phenyl-11*H*-dibenzo[f,h][1,2,4]triazolo[4′,3′:1,5]pyrazolo[3,4-c]cinnolin-13-amine 6, 13-phenyl-11H-dibenzo[f,h][1,2,4]triazolo[4′,3′:1,5]pyrazolo[3,4-c]cinnoline 7 and 11,14-dihydro-13H-dibenzo[f,h][1,2,4]triazolo[4′,3′:1,5]pyrazolo[3,4-*c*]cinnolin-13-one **8**, were tested for anti-inflammatory activity in Wistar and Sprague Dawley rats (the animals are described as mice in the Experimental section, obviously an error) [8]. Indomethacin was used as reference drug. The compounds were administered orally at a dose of 5 mg/kg body mass. The results are listed in Table 2. Compound **6** possesses weak anti-inflammatory activity, compound **8** shows intermediary activity, while compound **7** is almost as active as Indomethacin. The high anti-inflammatory activity of compound **7** was attributed to the presence of a secondary amine of triazole [triazine in the original paper] fragment.

The acute toxicity of compound **7** was determined in vivo in mice [8]. The compounds were administered by intraperitoneal injection. Indomethacin was used as standard drug. The results are displayed in Table 3. Compound **7** is moderately toxic, but less toxic than Indomethacin.

**Figure 4:**
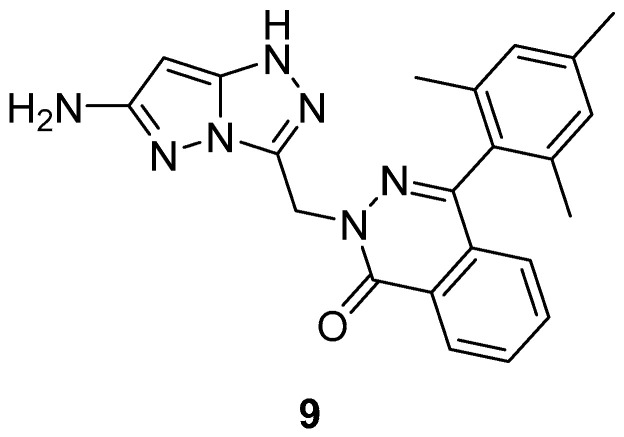
Chemical structure of compound **9**.

2-[(6-Amino-1*H*-pyrazolo[5,1-*c*][1,2,4]triazol-3-yl)methyl]-4-(2,4,6-trimethylphenyl)phthalazin-1(2*H*)-one **9** (Figure 4) was tested for anti-inflammatory activity in rats [9]. The control group (each group included 6 rats) was injected with 2% *w/v* acacia mucilage, while a second group was treated with Indomethacin as reference drug. The other seven groups were injected with the compound studied. The compounds were administered intraperitoneally at a dose of 1.5 mg/kg body mass. Then the rat paw oedema was induced with 0.1 mL *w/v* carrageenan subcutaneously. The results are listed in Table 4. Compound **9** possesses moderate anti-inflammatory activity.

## 4. Analgesic Activity

Compounds **6**–**8** (Figure 3) were tested for analgesic activity in Webster mice, with Valdecoxib as reference drug [8]. The compounds were administered subcutaneously at a dose of 5 mg/kg body mass. The results are listed in Table 5. Compound **6** showed intermediate analgesic activity, compound **7** possessed only weak activity, while compound **8** was very active, with its activity after 30 min becoming practically equal to that of Valdecoxib. The high analgesic activity of compound **8** was attributed to the presence of the secondary amine of the triazole fragment in its structure.

## 5. Antidiabetic Activity

**Figure 5:**
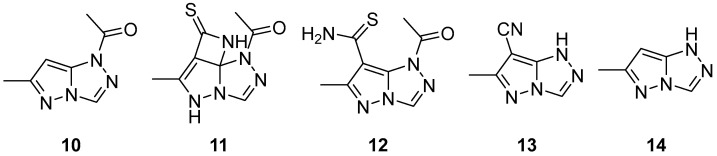
Chemical structures of compounds **10**–**14**.

Compounds **10**–**14** (Figure 5), 1-(6-methyl-1*H*-pyrazolo[5,1-*c*][1,2,4]triazol-1-yl)ethan-1-one **10**, 1-(6-methyl-7-thioxo-7,8-dihydro-1*H*,5*H*-azeto[3′,2′:4,5]pyrazolo[5,1-*c*][1,2,4]triazol-1-yl)ethan-1-one **11**, 1-acetyl-6-methyl-1*H*-pyrazolo[5,1-*c*][1,2,4]triazole-7-carbothioamide **12**, 6-methyl-1*H*-pyrazolo[5,1-*c*][1,2,4]triazole-7-carbonitrile **13**, and 6-methyl-1*H*-pyrazolo[5,1-*c*][1,2,4]triazole **14** (Figure 5) were tested for antidiabetic activity (*α*-glucosidase and *α*-amylase inhibitory activities) [10]. The antidiabetic drug Acarbose was used as reference. The results are shown in Table 6. It can be observed that compound **12** is the most potent inhibitor of *α*-glucosidase, while compounds **14** and **11** are potent inhibitors of *α*-amylase. Molecular docking studies were also reported in this paper.

## 6. Antibacterial Activity

**Figure 6:**
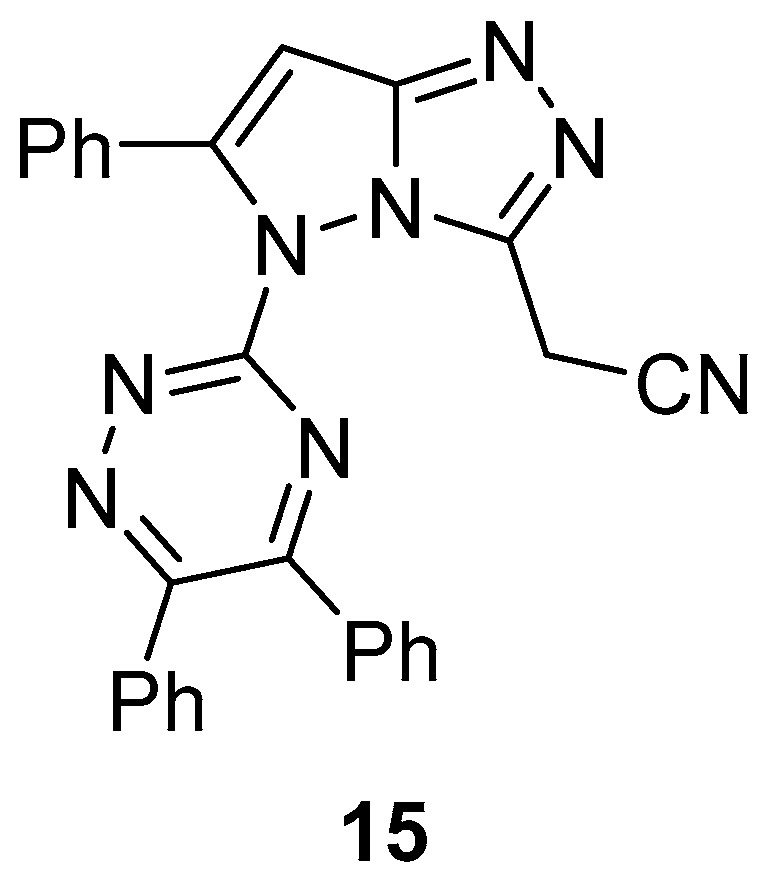
Chemical structure of compound **15**.

2-[5-(5,6-Diphenyl-1,2,4-triazin-3-yl)-6-phenyl-5*H*-pyrazolo[5,1-*c*][1,2,4]triazol-3-yl]acetonitrile **15** (Figure 6) was tested in vitro for antibacterial activity against *Staphylococcus aureus*, *Bacillus subtilis*, *Escherichia coli*, and *Proteus vulgaris* and was found to be inactive in all cases [11].

**Figure 7:**
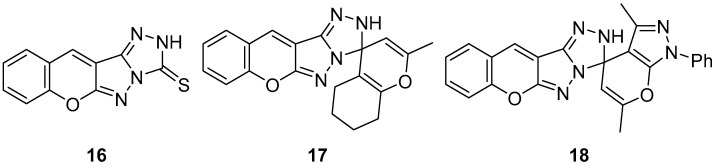
Chemical structures of compounds **16**–**18**.

In another study, three chromeno[2′,3′:3,4]pyrazolo[5,1-*c*][1,2,4]triazoles **16**–**18** (Figure 7), chromeno[2′,3′:3,4]pyrazolo[5,1-*c*][1,2,4]triazole-3(2*H*)-thione **16**, 2-methyl-5,6,7,8-tetrahydro-2′H-spiro[chromene-4,3′-chromeno[2′,3′:3,4]pyrazolo[5,1-*c*][1,2,4]triazole] **17**, and 3′,6′-dimethyl-1′-phenyl-1′*H*,2*H*-spiro[chromeno[2′,3′:3,4]pyrazolo[5,1-*c*][1,2,4]triazole-3,4′-pyrano[2,3-*c*]pyrazole] **18** were tested in vitro for antibacterial activity against two Gram-positive bacteria, *Bacillus cereus* and *Staphylococcus albus*, and two Gram-negative bacteria species, *Pseudomonas aeruginosa* and *E. coli* [12]. The results are displayed in Table 7. As can be seen, these compounds showed variable activity against bacteria.

The cytotoxicity of compounds **16**–**18** (Figure 7) was determined on brine shrimp (*Artemia salina*) larvae [12]. The results are shown in Table 8. Compound **17** has low toxicity, while compounds **16** and **18** are non-toxic.

**Figure 8:**
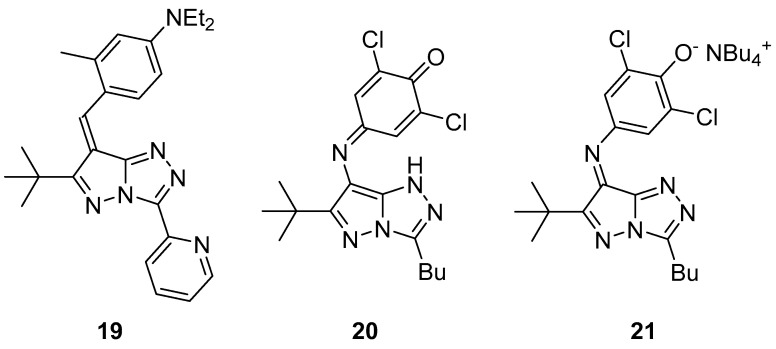
Chemical structures of compounds **19**–**21**.

Pyrazolo[5,1-*c*][1,2,4]triazoles **19**–**21** (Figure 8), 4-{[6-(1,1-dimethylethyl)-3-(pyridin-2-yl)-7*H*-pyrazolo[5,1-*c*][1,2,4]triazol-7-ylidene]methyl}-*N,N*-diethyl-3-methylaniline **19**, 4-{[3-butyl-6-(1,1-dimethylethyl)-1*H*-pyrazolo[5,1-*c*][1,2,4]triazol-7-yl]imino}-2,6-dichlorocyclohexa-2,5-dien-1-one **20**, and tetrabutylammonium 4-{[3-butyl-6-(1,1-dimethylethyl)-7*H*-pyrazolo[5,1-*c*][1,2,4]triazol-7-ylidene]amino}-2,6-dichlorophenolate **21** were tested for antibacterial activity against *Salmonella typhimurium* TA98 and TA100 [13]. H-1 [Kathon biocide; a 3:1 mixture of 5-chloro-2-methylisothiazol-3(2*H*)-one and 2-methylisothiazol-3(2*H*)-one)] was used as reference. The results are shown in Table 9 and Table 10. It can be seen that compounds **19**–**21** have antibacterial activity, while, at the same time, they are less toxic than the reference antibacterial agent (no increase in the number of revertant colonies was observed).

**Figure 9:**
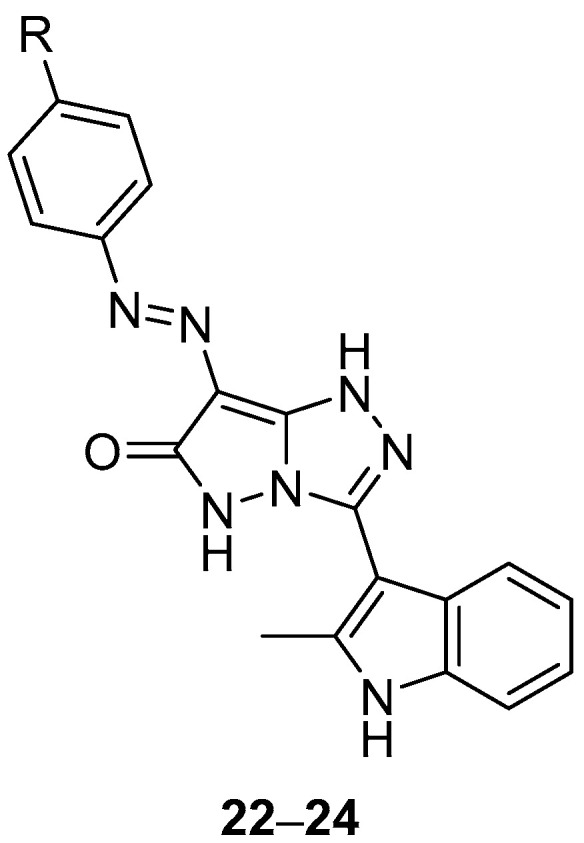
Chemical structures of compounds **22**–**24**. **22**: R = H; **23**: R = Me; **24**: R = Cl.

Pyrazolo[5,1-*c*][1,2,4]triazol-6(5*H*)-ones **22**–**24** (Figure 9), 3-(2-methyl-1*H*-indol-3-yl)-7-(phenyldiazenyl)-1*H*-pyrazolo[5,1-*c*][1,2,4]triazol-6(5*H*)-one **22**, 3-(2-methyl-1*H*-indol-3-yl)-7-(4-methylphenyldiazenyl)-1*H*-pyrazolo[5,1-*c*][1,2,4]triazol-6(5*H*)-one **23** and 7-(4-chlorophenyldiazenyl)-3-(2-methyl-1*H*-indol-3-yl)-1*H*-pyrazolo[5,1-*c*][1,2,4]triazol-6(5*H*)-one **24**, were also tested in vitro for antibacterial activity against four bacteria, *S. aureus*, *P. aeruginosa*, *B. subtilis* and *E. coli* [14]. Chloramphenicol was used as reference under the same conditions. The results are displayed in Table 11. Compound **23** exhibited the highest degree of inhibition against *P. aeruginosa* and *E. coli*, while compound **22** had a high inhibition effect against *P. aeruginosa*. Compound **24** showed a high degree of inhibition against *E. coli*. However, the activities of the tested compounds are much lower than those of the standard antibacterial agent used.

**Figure 10:**
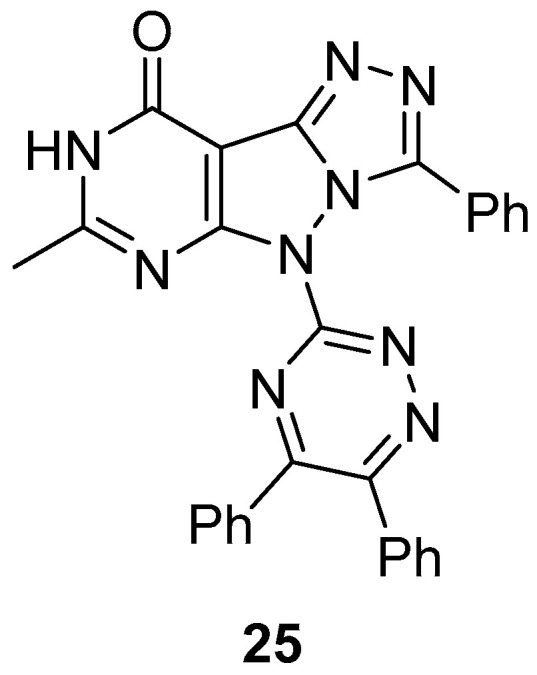
Chemical structure of compound **25**.

5-(5,6-Diphenyl-1,2,4-triazin-3-yl)-7-methyl-3-phenyl-5H-[1,2,4]triazolo[4′,3′:1,5]pyrazolo[3,4-*d*]pyrimidin-9(8*H*)-one **25** (Figure 10) was tested in vitro for antibacterial activity against three bacteria, *S. aureus* (MTCCB 737), *Staphylococcus epidermidis* (MTCCB 1824), and *E. coli* (MTCCB 1652) [15]. Tetracycline was used as standard drug against bacterial strains at 30 μg/mL concentration. The results are shown in Table 12. It can be noted that compound **25** showed good inhibition activities against all species of bacterial strains with respect to Tetracycline. The good antibacterial activity was attributed to the presence of the pyrazolo[3,4-*b*]pyrimidine scaffold fused with the bioactive heterocyclic moiety of 1,2,4-triazole. The minimum inhibitory concentrations (MIC, μg/mL) of compound **25** against *S. aureus* (MTCCB 737) and *E. coli* (MTCCB 1652) are shown in Table 13.

Compound **25** was also tested for cytotoxicity against *A. salina* larvae [15]. Bleomycin and gallic acid were used as standards. As can be seen in Table 14, compound **25** showed low toxicity against *A. salina* larvae.

In another study, compounds **6** and **8** (Figure 3) were tested in vitro for antibacterial activity against the same three bacteria, *S. aureus*, *S. epidermidis*, and *E. coli* [8]. The same standard as above was used (Tetracycline at 30 μg/mL concentration). The results are shown in Table 15. It can be seen that compound **8** showed good inhibition against all the species of bacteria, while compound **6** showed weak inhibition.

**Figure 11:**
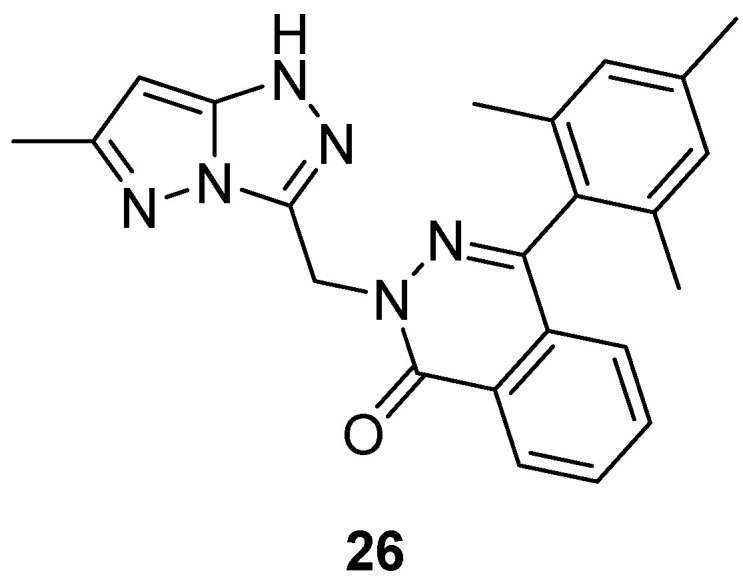
Chemical structure of compound **26**.

Compound **9** (Figure 4) and its analog 2-[(6-methyl-1*H*-pyrazolo[5,1-*c*][1,2,4]triazol-3-yl)methyl]-4-(2,4,6-trimethylphenyl)phthalazin-1(2*H*)-one **26** (Figure 11) were tested in vitro for antibacterial activity against four bacterial strains, *E. coli*, *S. aureus*, *B. subtilis* and *Salmonella typhi* [9,16]. Amoxicillin was used as standard drug. The observed activities are given in Table 16. The results indicate that compound **9** exhibited good antibacterial activity against *S. aureus*, *B. subtilis*, and *S. typhi* and lower activity against *E. coli*, while compound **26** showed weak activity against all four bacterial species.

**Figure 12:**
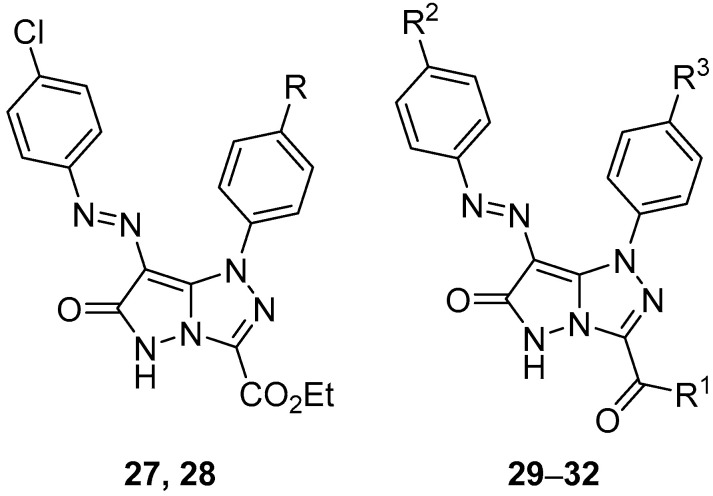
Chemical structures of compounds **27**–**32**. **27**: R = H; **28**: R = Me; **29**–**31**: R^1^ = Me; **29**: R^2^ = Cl**,** R^3^ = H; **30**: R^2^ = Cl, R^3^ = OMe; **31**: R^2^ = R^3^ = Me; **32**: R^1^ = Ph, R^2^ = Me, R^3^ = H.

In another study, six pyrazolo[5,1-*c*][1,2,4]triazoles **27**–**32** (Figure 12), ethyl 7-[(4-chlorophenyl)diazenyl]-6-oxo-1-phenyl-5,6-dihydro-1*H*-pyrazolo[5,1-*c*][1,2,4]triazole-3-carboxylate **27**, ethyl 7-[(4-chlorophenyl)diazenyl]-1-(4-methylphenyl)-6-oxo-5,6-dihydro-1*H*-pyrazolo[5,1-*c*][1,2,4]triazole-3-carboxylate **28**, 3-acetyl-7-[(4-chlorophenyl)diazenyl]-1-phenyl-1*H*-pyrazolo[5,1-*c*][1,2,4]triazol-6(5*H*)-one **29**, 3-acetyl-7-[(4-chlorophenyl)diazenyl]-1-(4-methoxyphenyl)-1*H*-pyrazolo[5,1-*c*][1,2,4]triazol-6(5*H*)-one **30**, 3-acetyl-1-(4-methylphenyl)-7-(4-methylphenyldiazenyl)-1*H*-pyrazolo[5,1-*c*][1,2,4]triazol-6(5*H*)-one **31**, and 3-benzoyl-7-(4-methylphenyldiazenyl)-1-phenyl-1*H*-pyrazolo[5,1-*c*][1,2,4]triazol-6(5*H*)-one **32** were tested in vitro for antibacterial activity against two Gram-positive bacteria, *Streptococcus pneumoniae* and *B. subtilis*, and two Gram-negative bacteria species, *P. aeruginosa* and *E. coli* [17]. Ampicillin and Gentamicin were used as standard antibacterial agents for Gram-positive and Gram-negative bacteria, respectively. The results are displayed in Table 17. With the exception of compounds **27**, **29**, and **30,** which showed low activity against *B. subtilis*, and compound **28**, which revealed low activity against *S. pneumoniae*, the compounds tested showed no antibacterial activity.

**Figure 13:**
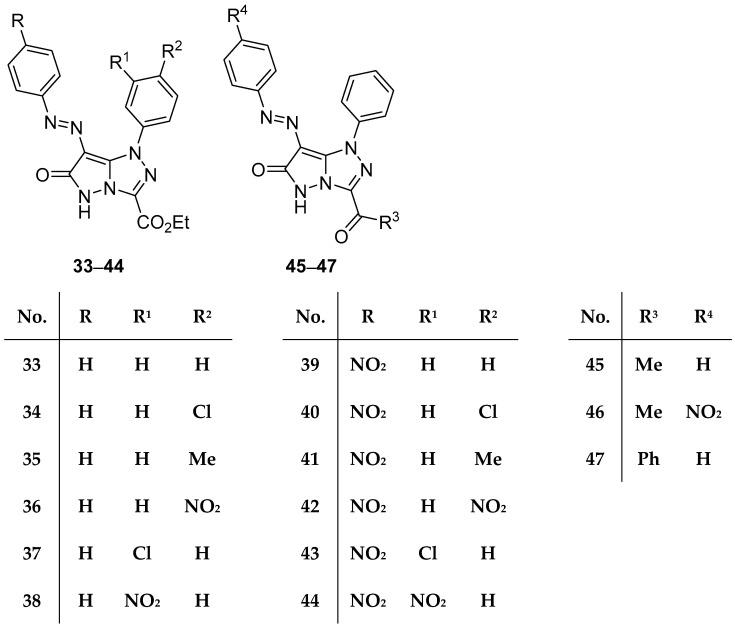
Chemical structures of compounds **33**–**47**.

In another study from the same laboratory, fifteen pyrazolo[5,1-*c*][1,2,4]triazoles **33**–**47** (Figure 13), ethyl 6-oxo-1-phenyl-7-(phenyldiazenyl)-5,6-dihydro-1*H*-pyrazolo[5,1-*c*][1,2,4]triazole-3-carboxylate **33**, ethyl 1-(4-chlorophenyl)-6-oxo-7-(phenyldiazenyl)-5,6-dihydro-1*H*-pyrazolo[5,1-*c*][1,2,4]triazole-3-carboxylate **34**, ethyl 1-(4-methylphenyl)-6-oxo-7-(phenyldiazenyl)-5,6-dihydro-1*H*-pyrazolo[5,1-*c*][1,2,4]triazole-3-carboxylate **35**, ethyl 1-(4-nitrophenyl)-6-oxo-7-(phenyldiazenyl)-5,6-dihydro-1*H*-pyrazolo[5,1-*c*][1,2,4]triazole-3-carboxylate **36**, ethyl 1-(3-chlorophenyl)-6-oxo-7-(phenyldiazenyl)-5,6-dihydro-1*H*-pyrazolo[5,1-*c*][1,2,4]triazole-3-carboxylate **37**, ethyl 1-(3-nitrophenyl)-6-oxo-7-(phenyldiazenyl)-5,6-dihydro-1*H*-pyrazolo[5,1-*c*][1,2,4]triazole-3-carboxylate **38**, ethyl 7-[(4-nitrophenyl)diazenyl]-6-oxo-1-phenyl-5,6-dihydro-1*H*-pyrazolo[5,1-*c*][1,2,4]triazole-3-carboxylate **39**, ethyl 1-(4-chlorophenyl)-7-[(4-nitrophenyl)diazenyl]-6-oxo-5,6-dihydro-1*H*-pyrazolo[5,1-*c*][1,2,4]triazole-3-carboxylate **40**, ethyl 1-(4-methylphenyl)-7-[(4-nitrophenyl)diazenyl]-6-oxo-5,6-dihydro-1*H*-pyrazolo[5,1-*c*][1,2,4]triazole-3-carboxylate **41**, ethyl 1-(4-nitrophenyl)-7-[(4-nitrophenyl)diazenyl]-6-oxo-5,6-dihydro-1*H*-pyrazolo[5,1-*c*][1,2,4]triazole-3-carboxylate **42**, ethyl 1-(3-chlorophenyl)-7-[(4-nitrophenyl)diazenyl]-6-oxo-5,6-dihydro-1*H*-pyrazolo[5,1-*c*][1,2,4]triazole-3-carboxylate **43**, ethyl 1-(3-nitrophenyl)-7-[(4-nitrophenyl)diazenyl]-6-oxo-5,6-dihydro-1*H*-pyrazolo[5,1-*c*][1,2,4]triazole-3-carboxylate **44**, 3-acetyl-1-phenyl-7-(phenyldiazenyl)-1*H*-pyrazolo[5,1-*c*][1,2,4]triazol-6(5*H*)-one **45**, 3-acetyl-7-[(4-nitrophenyl)diazenyl]-1-phenyl-1*H*-pyrazolo[5,1-*c*][1,2,4]triazol-6(5*H*)-one **46**, and 3-benzoyl-1-phenyl-7-(phenyldiazenyl)-1*H*-pyrazolo[5,1-*c*][1,2,4]triazol-6(5*H*)-one **47** were tested in vitro for antibacterial activity against the same four bacterial strains, using the same standards for reference [18]. The determined activities are included in Table 18. The results reveal that compounds **34**, **35**, **37**, **39**, **45**, and **46** have medium to weak activity against both Gram-positive bacteria, while compound **47** shows weak activity against *B. subtilis*. With the exception of compound **35**, which has medium activity against *E. coli*, the compounds tested showed no activity against Gram-negative bacteria. A molecular docking study was also included in the work.

**Figure 14:**
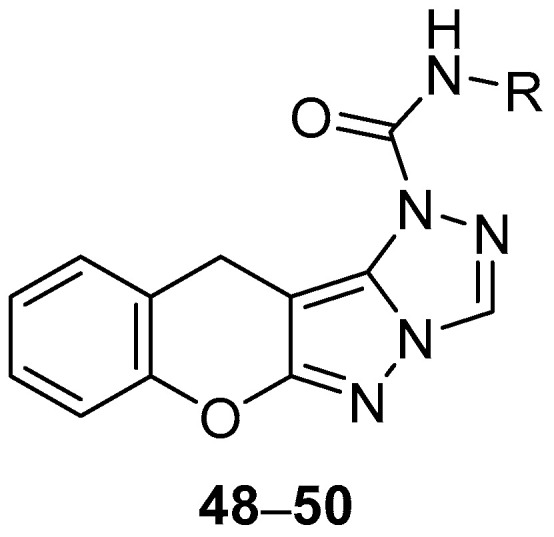
Chemical structures of compounds **48**–**50**. **48**: R = 2,4-F_2_-C_6_H_3_; **49**: R = Et; **50**: R = 2-F-4-Et-C_6_H_3_CH_2_.

A Chinese patent reports the in vitro testing of chromeno[2′,3′:3,4]pyrazolo[5,1-*c*][1,2,4]triazoles **48**–**50** (Figure 14), *N*-(2,4-difluorophenyl)chromeno[2′,3′:3,4]pyrazolo[5,1-*c*][1,2,4]triazole-1(11*H*)-carboxamide **48**, *N*-ethylchromeno[2′,3′:3,4]pyrazolo[5,1-*c*][1,2,4]triazole-1(11*H*)-carboxamide **49** and *N*-(4-ethyl-2-fluorobenzyl)chromeno[2′,3′:3,4]pyrazolo[5,1-*c*][1,2,4]triazole-1(11*H*)-carboxamide **50**, against *S. aureus*, with Penicillin as standard [19]. The results are shown in Table 19. Compounds **48** and **50**, both containing a fluorine-substituted benzene ring, show good inhibition against *S. aureus*, while the activity of compound **49** is significantly lower.

## 7. Antifungal Activity

Compound **15** (Figure 6) was also tested in vitro for antifungal activity against *Aspergillus niger* and *Penicillium chrysogenum* (formerly known as *P. notatum*) and was found to be inactive in all cases [11].

**Figure 15:**
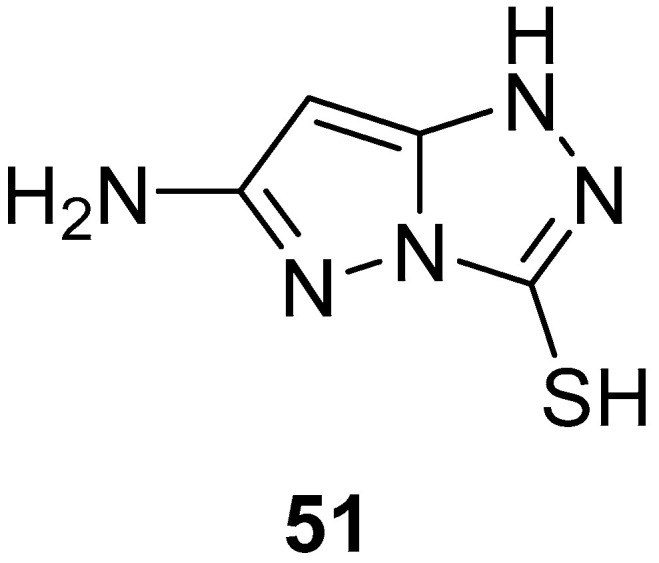
Chemical structure of compound **51**.

6-Amino-1*H*-pyrazolo[5,1-*c*][1,2,4]triazole-3-thiol **51** (Figure 15) was tested in vitro for antifungal activity against four fungal species, *Aspergillus ochraceus* Wilhelm (AUCC-230), *P. chrysogenum* Thom (AUCC-530), *Aspergillus flavus* Link (AUCC-164), and *Candida albicans* (Robim) Berkho (AUCC-1720) [20]. Mycostatin (30 μg/mL) was used as reference. [This compound is actually a tautomer form of compound **1**, reported as such in this paper.] The results are listed in Table 20. Compound **51** is nearly as active as Mycostatin against *A. ochraceus*, *P. chrysogenum*, and *A. flavus* (MIC values were 50–75 μg/mL) but less active against *C. albicans*.

Compounds **22**–**24** (Figure 9) were tested in vitro for antifungal activity against four fungal species, *Aspergillus fumigatus*, *Penicillium italicum*, *Syncephalastrum racemosum*, and *Candida albicans* [14]. The fungicide Terbinafin was used as reference. The results are displayed in Table 21. Compounds **22** and **23** exhibited the highest degree of inhibition against *A. fumigatus*; the rest of the activities were low. However, the activities of the tested compounds are much lower than that of the standard antifungal agent used.

Compound **25** (Figure 10) was tested in vitro for antifungal activity against three fungi, *A. fumigatus*, *Aspergillus niger*, and *Alternaria alternata* [15]. Ketoconazole was used as standard drug against fungal strains at 30 μg/mL concentration. The results are shown in Table 22. It can be noted that compound **25** showed good inhibition activities against *A. fumigatus* with respect to Ketoconazole, and lower inhibition against the other two species. The minimum inhibitory concentrations (MIC, μg/mL) of compound **25** against *A. niger* and *A. alternata* are shown in Table 23.

Compounds **6** and **8** (Figure 3) were tested in vitro for antifungal activity against the same three fungal species, *A. fumigatus*, *A. niger*, and *A. alternata* [8]. The same standard as above was used (Ketoconazole at 30 μg/mL concentration). The results are shown in Table 24. It can be seen that compound **8** shows good inhibition against all the species of fungi, while compound **6** displays weak inhibition.

Compound **26** (Figure 11) was tested in vitro for antifungal activity against two fungal strains, *A. niger* and *C. albicans* [16]. Again, Ketoconazole was used as standard drug (30 μg/mL concentration). The observed activities are given in Table 25. The results indicate low activities of compound **26** against both fungal strains.

Compound **9** (Figure 4) was subjected to the same tests as its analog **26** above [9]. The determined activities are reported in Table 26. This compound exhibited moderate activity against *A. niger* and low activity against *C. albicans*.

When compounds **27**–**32** (Figure 12) were tested in vitro for antifungal activity against four fungal species, *A. fumigatus*, *S. racemosum*, *Geotricum candidum*, and *C. albicans*, using Amphotericin B as standard antifungal agent, they showed no antifungal activity at all [17].

Compounds **33**–**47** (Figure 13), when tested under the same conditions, also showed no antifungal activity at all [18].

## 8. Antiviral Activity

**Figure 16:**
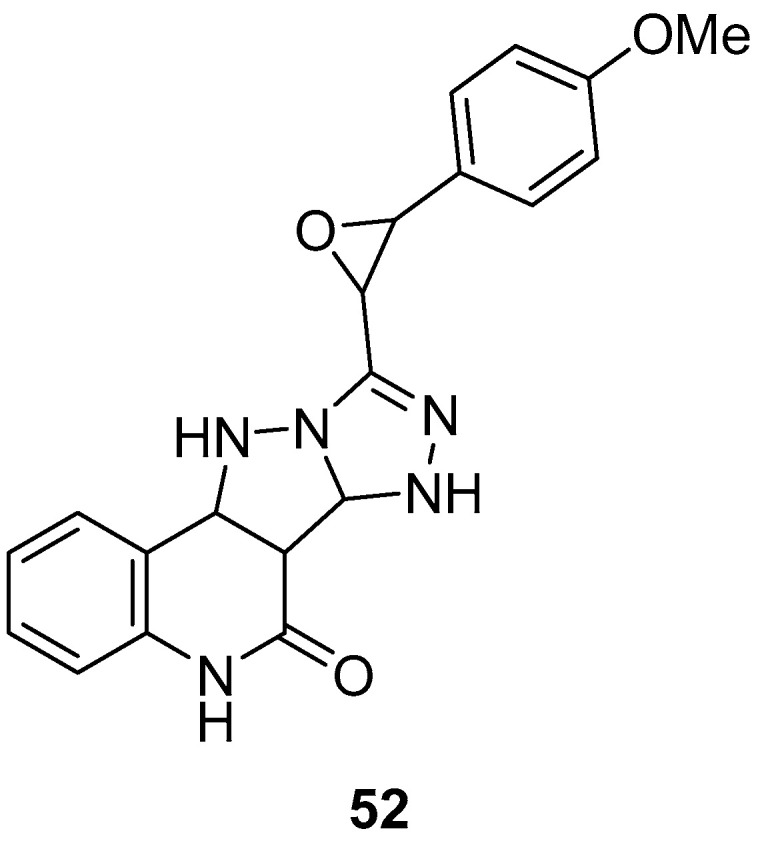
Chemical structure of compound **52**.

9-[3-(4-Methoxyphenyl)oxiran-2-yl]-6b,7,11,11a-tetrahydro-5H-[1,2,4]triazolo[4′,3′:1,5]pyrazolo[4,3-*c*]quinolin-6(6a*H*)-one **52** (Figure 16) was tested in vitro for antiviral activity against infectious bursal disease virus (IBDV) in specific pathogen-free (SPF) chicken embryos, with un-inoculated SPF eggs as control of embryo [21]. Ribavirin was used as reference drug. The results are shown in Table 27. Compound **52** displayed weak activity against IBDV.

## 9. Antiprotozoal Activity

**Figure 17:**
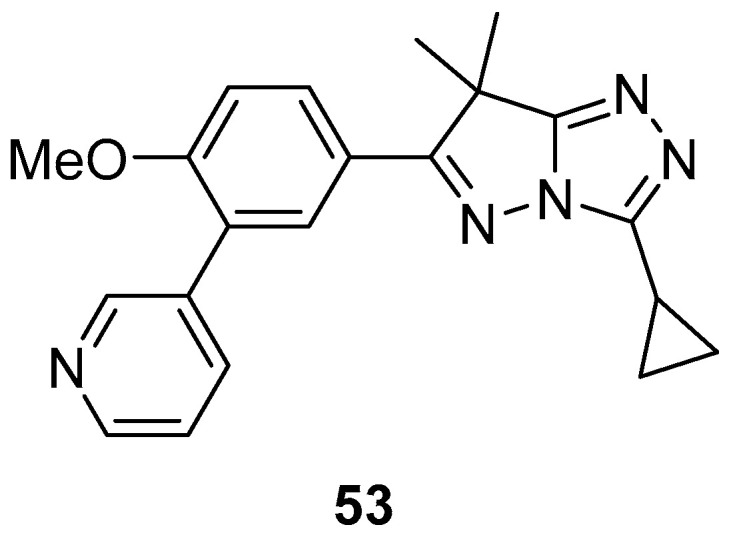
Chemical structure of compound **53**.

3-Cyclopropyl-6-[4-methoxy-3-(pyridin-3-yl)phenyl]-7,7-dimethyl-7H-pyrazolo[5,1-*c*][1,2,4]triazole **53** (Figure 17) was tested for antiprotozoal activity (phenotypic activity against intracellular amastigotes) against the causative agent of Chagas disease, *Trypanosoma cruzi* [22]. The results are shown in Table 28. The pIC_50_ value of **53** indicates a moderate activity compared with that of the lead compound, NPD-0227 (2-isopropyl-5-[4-methoxy-3-(pyridin-3-yl)phenyl]-4,4-dimethyl-2,4-dihydro-3*H*-pyrazol-3-one) [23] (6.4).

## 10. Anticancer Activity

**Figure 18:**
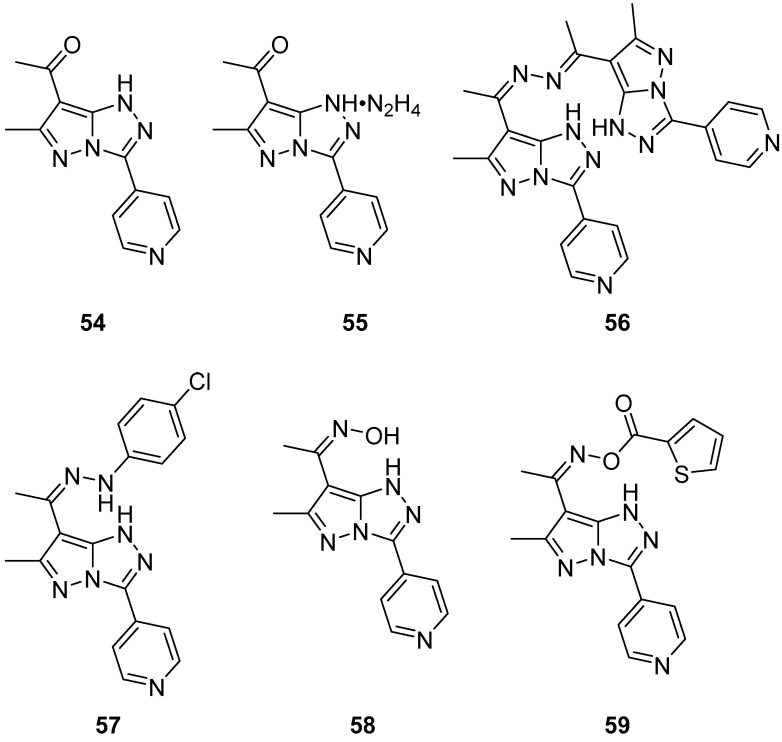
Chemical structures of compounds **54**–**59**.

3-(Pyridin-4-yl)-1*H*-pyrazolo[5,1-*c*][1,2,4]triazoles **54**–**59**, 1-[6-methyl-3-(pyridin-4-yl)-1*H*-pyrazolo[5,1-*c*][1,2,4]triazol-7-yl]ethan-1-one **54**, its hydrazinium salt **55**, 1,2-bis{1-[6-methyl-3-(pyridin-4-yl)-1*H*-pyrazolo[5,1-*c*][1,2,4]triazol-7-yl]ethylidene}hydrazine **56**, 7-{1-[2-(4-chlorophenyl)hydrazinylidene]ethyl}-6-methyl-3-(pyridin-4-yl)-1*H*-pyrazolo[5,1-*c*][1,2,4]triazole **57**, 1-[6-methyl-3-(pyridin-4-yl)-1*H*-pyrazolo[5,1-*c*][1,2,4]triazol-7-yl]ethan-1-one oxime **58**, and 1-[6-methyl-3-(pyridin-4-yl)-1*H*-pyrazolo[5,1-*c*][1,2,4]triazol-7-yl]ethan-1-one *O*-thiophene-2-carbonyl oxime **59** (Figure 18) were tested in vitro for cytotoxicity against Ehrlich–Lettre ascites carcinoma (EAC) tumor cells, with Doxorubicin as reference [24]. The results are displayed in Table 29. Compounds **56** and **57** proved to be active toward the used tumor cells; compounds **54**, **55**, and **58** showed moderate activities, while compound **59** showed no activity, possibly because of its low solubility in the culture medium.

The activity of compounds **56** and **57** against a liver carcinoma cell line (Hep G2) was also examined, again with Doxorubicin as reference [24]. The results are shown in Table 30. The low IC_50_ value of compound **57** is an indicator of its high inhibitory activity against Hep G2.

**Figure 19:**
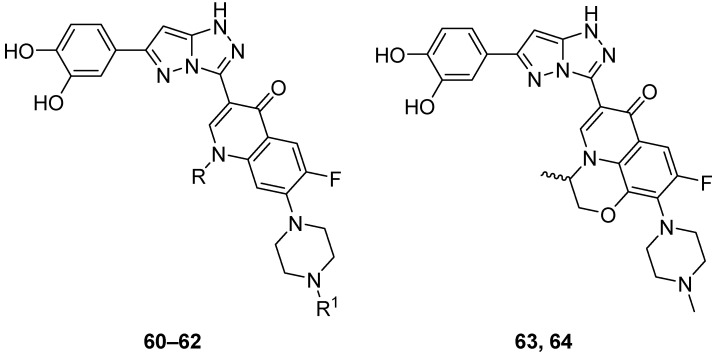
Chemical structures of compounds **60**–**64**. **60**: R = Et, R^1^ = H; **61, 62**: R = cyclopropyl; **61**: R^1^ = H; **62**: R^1^ = Et.

The in vitro antitumor activity for five fluoroquinolones **60**–**64** (Figure 19), 3-[6-(3,4-dihydroxyphenyl)-1*H*-pyrazolo[5,1-*c*][1,2,4]triazol-3-yl]-6-fluoro-1-methyl-7-(piperazin-1-yl)quinolin-4(1*H*)-one **60**, 1-cyclopropyl-3-[6-(3,4-dihydroxyphenyl)-1*H*-pyrazolo[5,1-*c*][1,2,4]triazol-3-yl]-6-fluoro-7-(piperazin-1-yl)quinolin-4(1*H*)-one **61**, 1-cyclopropyl-3-[6-(3,4-dihydroxyphenyl)-1*H*-pyrazolo[5,1-*c*][1,2,4]triazol-3-yl]-7-(4-ethylpiperazin-1-yl)-6-fluoroquinolin-4(1*H*)-one **62**, racemic 6-[6-(3,4-dihydroxyphenyl)-1*H*-pyrazolo[5,1-*c*][1,2,4]triazol-3-yl]-9-fluoro-3-methyl-10-(4-methylpiperazin-1-yl)-2,3-dihydro-7H-[1,4]oxazino[2,3,4-*ij*]quinolin-7-one **63**, and its *S* enantiomer **64** against L1210 (murine leukemia) and CHO (Chinese hamster ovary) cell lines was evaluated via their respective IC_50_ values [25]. The results are summarized in Table 31. While the five parent fluoroquinolone antibiotics (norfloxacin, ciprofloxacin, enrofloxacin, ofloxacin, and levofloxacin) had poor inhibitory activities against these cancer line cells (IC_50_ > 150 μmol/L), compounds **60**–**64** showed IC_50_ values < 10 μmol/L, with compounds **61** and **64** being the most active.

**Figure 20:**
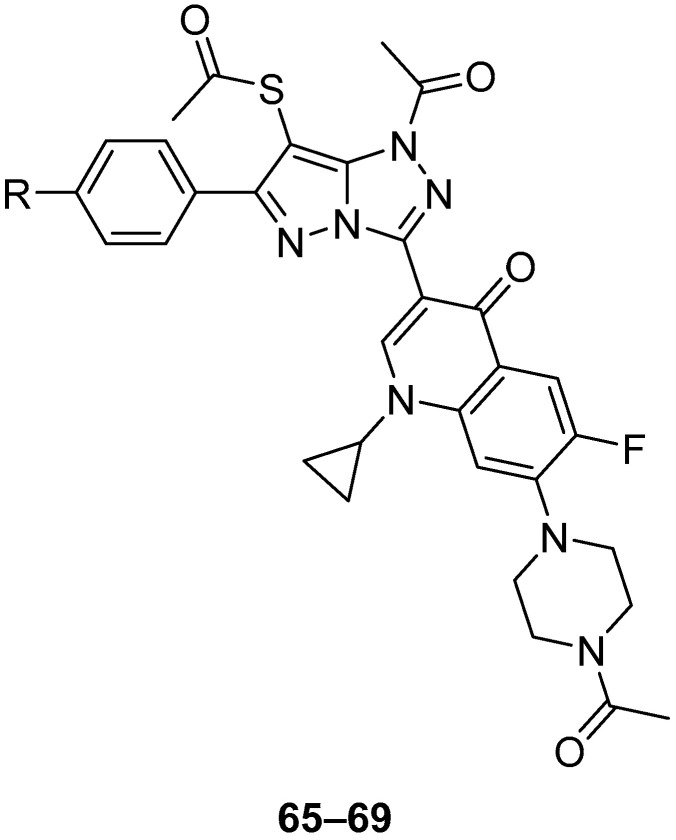
Chemical structures of compounds **65**–**69**. **65**: R = H; **66**: R = OMe; **67**: R = Me; **68**: R = Cl; **69**: R = NO_2_.

In another study, another five fluoroquinolones **65**–**69** (Figure 20), *S*-{1-acetyl-3-[7-(4-acetylpiperazin-1-yl)-1-cyclopropyl-6-fluoro-4-oxo-1,4-dihydroquinolin-3-yl]-6-phenyl-1*H*-pyrazolo[5,1-*c*][1,2,4]triazol-7-yl} ethanethioate **65**, *S*-{1-acetyl-3-[7-(4-acetylpiperazin-1-yl)-1-cyclopropyl-6-fluoro-4-oxo-1,4-dihydroquinolin-3-yl]-6-(4-methoxyphenyl)-1*H*-pyrazolo[5,1-*c*][1,2,4]triazol-7-yl} ethanethioate **66**, *S*-{1-acetyl-3-[7-(4-acetylpiperazin-1-yl)-1-cyclopropyl-6-fluoro-4-oxo-1,4-dihydroquinolin-3-yl]-6-(4-methylphenyl)-1*H*-pyrazolo[5,1-*c*][1,2,4]triazol-7-yl} ethanethioate **67**, *S*-{1-acetyl-3-[7-(C)-1-cyclopropyl-6-fluoro-4-oxo-1,4-dihydroquinolin-3-yl]-6-(4-chlorophenyl)-1*H*-pyrazolo[5,1-*c*][1,2,4]triazol-7-yl} ethanethioate **68**, and *S*-[1-acetyl-3-[7-(4-acetylpiperazin-1-yl)-1-cyclopropyl-6-fluoro-4-oxo-1,4-dihydroquinolin-3-yl]-6-(4-nitrophenyl)-1*H*-pyrazolo[5,1-*c*][1,2,4]triazol-7-yl} ethanethioate **69** were tested in vitro for antitumor activity against L1210 (murine leukemia), HL-60 (human leukemia) and CHO (Chinese hamster ovary) cell lines, with Ciprofloxacin as standard [26]. The results are given in Table 32. All these compounds showed IC_50_ values in the micromolar range, with compound **69** being the most active against all three cell lines, while Ciprofloxacin showed much weaker activity. The structure–activity relationships from these two studies [25,26] show that by substituting the 3-carboxyl group in fluoroquinolones with a fused heterobicycle is conducive to antitumor activity, and that the pyrazolo[5,1-*c*][1,2,4]triazoles investigated have greater antitumor activity than their triazolo[3,4-b][1,3,4]thiadiazine precursors and, in the case of compounds **65**–**69**, the latter’s 4-acetylpiperazinyl analogs.

**Figure 21:**
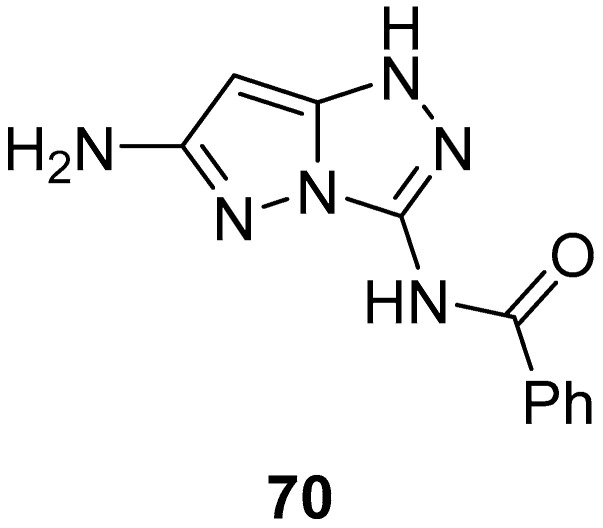
Chemical structure of compound **70**.

*N*-(6-Amino-1*H*-pyrazolo[5,1-*c*][1,2,4]triazol-3-yl)benzamide **70** (Figure 21) was tested in vitro for antitumor activity against a panel of four human tumor cell lines: hepatocellular carcinoma Hep G2, lung fibroblasts WI 38, kidney of a normal adult African green monkey VERO, and breast cancer MCF-7 [27]. 5-Fluorouracil was used as reference. The results are reported in Table 33. As can be observed, compound **70** showed weak inhibitory activity against all four cell lines.

**Figure 22:**
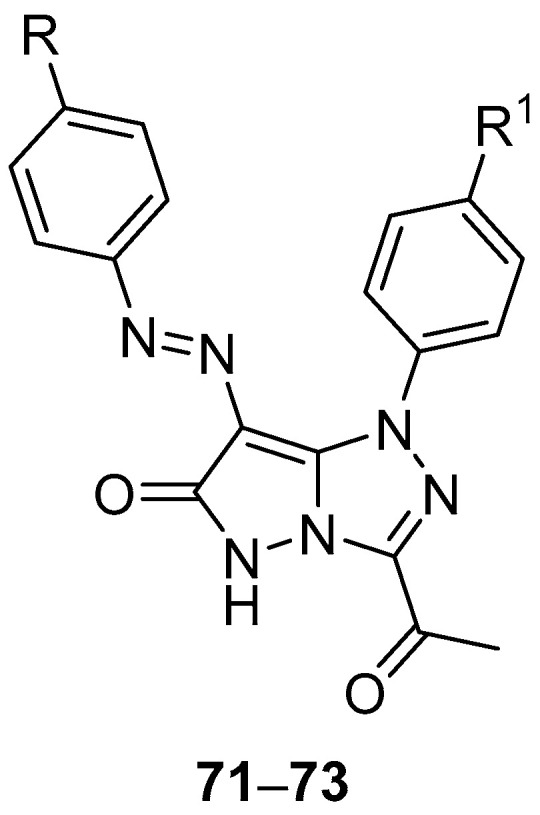
Chemical structures of compounds **71**–**73**. **71**: R = R^1^ = Cl; **72, 73**: R = Me; **72**: R^1^ = Cl; **73**: R^1^ = OMe.

Pyrazolo[5,1-*c*][1,2,4]triazoles **27**–**32** (Figure 12) and **71**–**73** (Figure 22), 3-acetyl-1-(4-chlorophenyl)-7-[(4-chlorophenyl)diazenyl]-1*H*-pyrazolo[5,1-*c*][1,2,4]triazol-6(5*H*)-one **71**, 3-acetyl-1-(4-chlorophenyl)-7-[(4-methylphenyl)diazenyl]-1*H*-pyrazolo[5,1-*c*][1,2,4]triazol-6(5*H*)-one **72**, and 3-acetyl-1-(4-methoxyphenyl)-7-[(4-methylphenyl)diazenyl]-1*H*-pyrazolo[5,1-*c*][1,2,4]triazol-6(5*H*)-one **73** were tested in vitro for antitumor activity against hepatocellular carcinoma Hep G2 and colon cancer HCT116 cell lines, with 5-fluorouracil, Doxorubicin and Imatinib as standard drugs [17]. The results are summarized in Table 34. Some of the tested compounds showed good activity against both cancer cell lines: the most active compounds against Hep G2 hepatocellular carcinoma cells were compounds **27**, **29**, and **72**, while compounds **28**, **71**, and **72** were the most active against colon cancer HCT116 cells.

**Figure 23:**
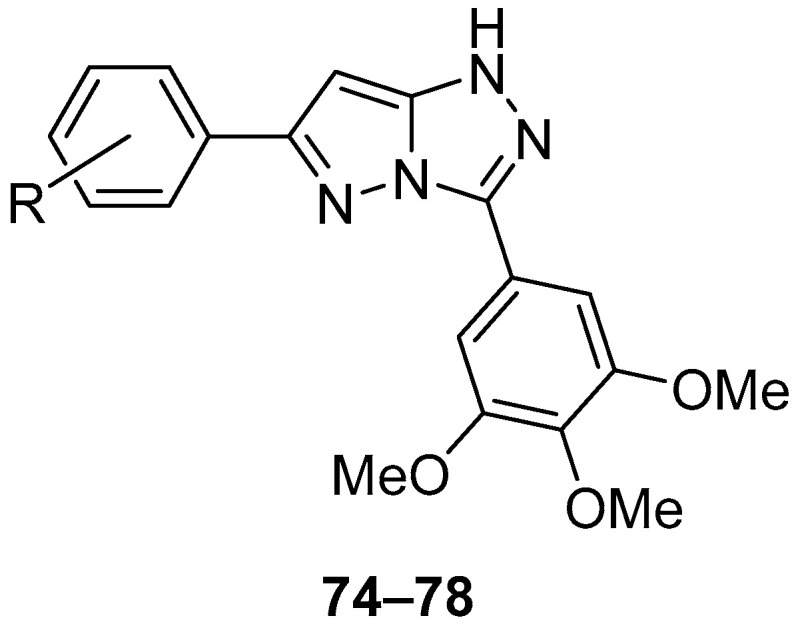
Chemical structures of compounds **74**–**78**. **74**: R = 4-Me; **75**: R = 4-Cl; **76**: R = 4-OMe; **77**: R = 3-NO_2_-4-OMe-; **78**: R = 3-NH_2_-4-OMe.

Compounds **74**–**78** (Figure 23), 6-(4-methylphenyl)-3-(3,4,5-trimethoxyphenyl)-1*H*-pyrazolo[5,1-*c*][1,2,4]triazole **74**, 6-(4-chlorophenyl)-3-(3,4,5-trimethoxyphenyl)-1*H*-pyrazolo[5,1-*c*][1,2,4]triazole **75**, 6-(4-methoxyphenyl)-3-(3,4,5-trimethoxyphenyl)-1*H*-pyrazolo[5,1-*c*][1,2,4]triazole **76**, 6-(4-methoxy-3-nitrophenyl)-3-(3,4,5-trimethoxyphenyl)-1*H*-pyrazolo[5,1-*c*][1,2,4]triazole **77**, and 2-methoxy-5-[3-(3,4,5-trimethoxyphenyl)-1*H*-pyrazolo[5,1-*c*][1,2,4]triazol-6-yl]aniline **78** were tested in vitro for antitumor activity against human gastric adenocarcinoma SGC-7901, human oral epithelial cancer KB, and human fibrosarcoma HT1080 cell lines, with Combretastatin A-4 and Doxorubicin as standard drugs [28]. The results are presented in Table 35. All the tested compounds showed good inhibition of all three cancer cell lines studied, with compounds **75** and **78** being the most active.

The acute toxicity of compounds **75** and **76** (Figure 23) was determined in vivo in mice at a dose of 500 mg/kg [28]. The compounds were administered by intraperitoneal injection. Since all the mice survived and returned to normal after the administration of this compound stopped, the LD_50_ value for intraperitoneal administration was considered greater than 500 mg/kg.

The most active compounds in vitro, **75** and **78**, were also investigated in vivo on S-180 sarcoma model mice, using 5-fluorouracil as reference [28]. The results are presented in Table 36.

**Figure 24:**
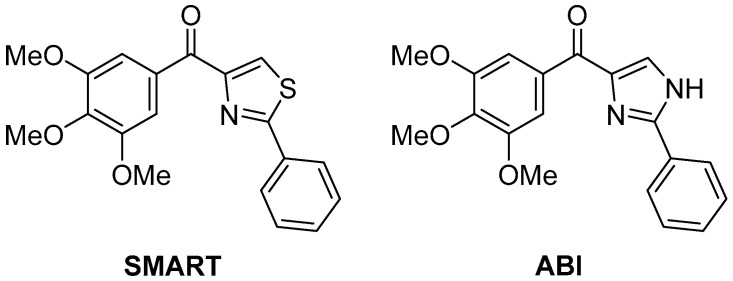
Chemical structures of compounds **SMART** and **ABI**.

The same compounds **74**–**78** were later tested again in vitro for antiproliferative activity against human gastric adenocarcinoma SGC-7901, human lung adenocarcinoma A549, and human fibrosarcoma HT1080 cell lines [29], with two compounds with potent antiproliferative activity, **SMART** [30] [(2-phenylthiazol-4-yl)(3,4,5-trimethoxyphenyl)methanone] and **ABI** [31] [(2-phenyl-1*H*-imidazol-4-yl)(3,4,5-trimethoxyphenyl)methanone] (Figure 24) as positive controls. The results are displayed in Table 37. It can be observed that all the pyrazolo[5,1-*c*][1,2,4]triazoles tested showed modest antiproliferative activity, especially in comparison with SMART. A number of 1H-[1,2,4]triazolo[3,4-b][1,3,4]thiadiazole analogs were also tested in this study, and several of these compounds showed potent antiproliferative activity at sub-micromolar or nanomolar concentrations against the three different cancer cell lines; one derivative in particular, the analog of **78**, showed activities close to those of SMART (0.022 ± 0.006, 0.029 ± 0.011, 0.027 ± 0.013, respectively).

Compounds **33**–**47** (Figure 13) were tested in vitro for antitumor activity against Hep G2 and HCT116 cell lines, using 5-fluorouracil, Doxorubicin, and Imatinib as standard drugs [18]. The results are shown in Table 38. These compounds showed weak to moderate activity against both cell lines, the most active being compounds **43** and **47**.

**Figure 25:**
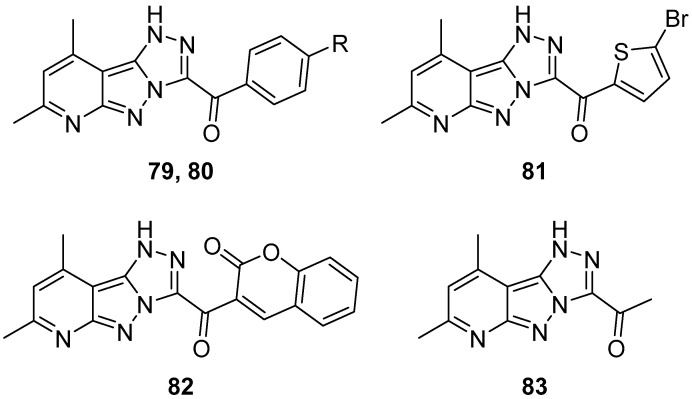
Chemical structures of compounds **79**–**83**. **79**: R = F; **80**: R = Cl.

1*H*-[1,2,4]Triazolo[4′,3′:1,5]pyrazolo[3,4-*b*]pyridines **79**–**83**: (7,9-dimethyl-1*H*-[1,2,4]triazolo[4′,3′:1,5]pyrazolo[3,4-*b*]pyridin-3-yl)(4-fluorophenyl)methanone **79**, (4-chlorophenyl)(7,9-dimethyl-1*H*-[1,2,4]triazolo[4′,3′:1,5]pyrazolo[3,4-*b*]pyridin-3-yl)methanone **80**, (5-bromothiophen-2-yl)(7,9-dimethyl-1*H*-[1,2,4]triazolo[4′,3′:1,5]pyrazolo[3,4-*b*]pyridin-3-yl)methanone **81**, 3-(7,9-dimethyl-1*H*-[1,2,4]triazolo[4′,3′:1,5]pyrazolo[3,4-*b*]pyridine-3-carbonyl)-2*H*-chromen-2-one **82**, and 1-(7,9-dimethyl-1*H*-[1,2,4]triazolo[4′,3′:1,5]pyrazolo[3,4-*b*]pyridin-3-yl)ethan-1-one **83** (Figure 25) were tested in vitro for antitumor activity against HCT116, Hep G2, HeLa (cervical cancer) and MCF-7 cell lines, with Doxorubicin as reference [32]. The results are listed in Table 39. Compound **82** exhibited very good activity against all cell lines, while all the other compounds demonstrated weak activity in all cases. The remarkable activity of compound **82** could be attributed to the presence of the coumarin fragment in its molecule.

Later, in a study on the discovery of 1,2,4-triazole-based inhibitors of aromatase (CYP19A1), for the treatment of hormone receptor (HR)-positive breast cancer, compounds **79**–**82** were among the 78 compounds used to generate the pharmacophore model [33]. Compounds **80** and **81** were included in the training set (39 compounds), and compounds **79** and **82** in the test set (39 compounds). In the end, this study led to two 1,2,4-triazole-based structures with better estimated activity and fit value than the standard drug Letrozole.

**Figure 26:**
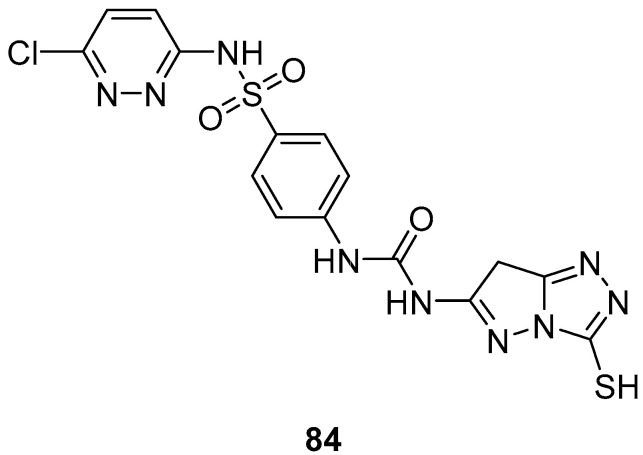
Chemical structure of compound **84**.

*N*-(4-{[(6-Chloropyridazin-3-yl)amino]sulfonyl}phenyl)-*N*′-(3-sulfanyl-7*H*-pyrazolo[5,1-*c*][1,2,4]triazol-6-yl)urea **84** (Figure 26) was subjected to an in vitro cytotoxicity screening against 21 cancer cell lines, representing eight subpanels: leukemia (CCRF-CEM and SR), non-small-cell lung (EKVX, HOP-62, HOP-92 and NCI-H522), central nervous system (SF-268 and SNB-75), melanoma (UACC-62), ovarian (IGROV1 and SK-OV-3), renal (A498, CAK-1 and UO-31), prostate (PC-3), and breast (MCF7, MDA-MB231/ATCC, HS 578T, BT-549, T-47D, and MDA-MB-468), at a single dose of 10^−5^ M (10 μM) [34]. The results were reported as the % growth inhibition (GI) against the cell lines. With the exception of the leukemia SR cell line, against which compound **84** displayed minimal activity (GI = 10%), and renal cancer UO-31 cell line (GI = 14%), no inhibitory activities of this compound were observed (GI < 10%).

## 11. C3a Receptor Binding Activity

**Figure 27:**
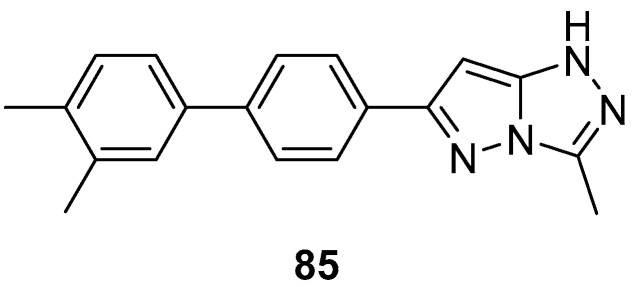
Chemical structure of compound **85**.

6-(3′,4′-Dimethyl-[1,1′-biphenyl]-4-yl)-3-methyl-1*H*-pyrazolo[5,1-*c*][1,2,4]triazole **85** (Figure 27) showed good C3a receptor binding activity, with an IC_50_ value of 71 nM [35].

## 12. Miscellaneous Biological Activities

**Figure 28:**
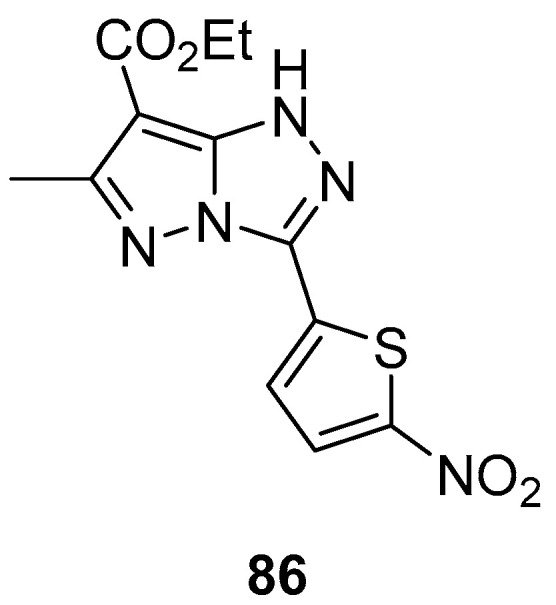
Chemical structure of compound **86**.

An in silico screen of 1.1 million compounds was performed, using three-dimensional E47-Id1 interaction mapping to identify small molecules that could potentially inhibit the E47-Id1 interaction [36]. Ethyl 6-methyl-3-(5-nitrothiophen-2-yl)-1*H*-pyrazolo[5,1-*c*][1,2,4]triazole-7-carboxylate **86** (Figure 28) was among the 364 structures identified by this screen, but not among the compounds showing the most pronounced anti-Id activity in the gel shift assay.

## 13. Cytotoxicity

**Figure 29:**
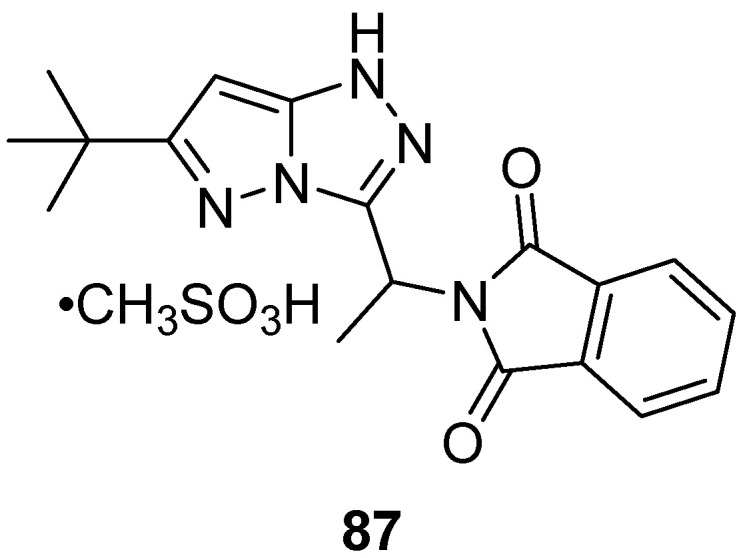
Chemical structure of compound **87**.

2-{1-[6-(1,1-Dimethylethyl)-1*H*-pyrazolo[5,1-*c*][1,2,4]triazol-3-yl]ethyl}-1*H*-isoindole-1,3(2*H*)-dione hydromethanesulfonate (mesylate) **87** (Figure 29) was tested for toxicity in rats by oral administration [37]. In contrast to the corresponding hydrochloride, which caused strong skin irritation, salt **87** had an acute toxicity of 2000 mg/kg or more and caused no skin irritation, mutagenicity, or rash.

## 14. Ulcerogenic Activity

Compound **7** (Figure 3) was also tested for ulcerogenic activity in rats at dose levels of 10, 50, and 100 mg/kg body mass [8]. Indomethacin was used as reference drug. The compounds were administered per os. The results are shown in Table 40. It can be observed that compound **8** possesses no ulcerogenic activity.

## 15. Conclusions

A number of studies on the biological activities of various pyrazolo[5,1-*c*][1,2,4]triazoles have been conducted and published, including several patents. Although the number of pyrazolo[5,1-*c*][1,2,4]triazoles studied as drug candidates so far is not very great (<100) compared with other scaffolds, this moiety is a promising motif for future drug discovery and development projects. Significantly, the pyrazolo[5,1-*c*][1,2,4]triazoles investigated so far have shown little or no toxicity. One obvious direction is that of synthetic nucleoside analogs, with the pyrazolo[5,1-*c*][1,2,4]triazole scaffold as a good isostere for purine. There are many methods developed for the synthesis and further functionalization of pyrazolo[5,1-*c*][1,2,4]triazoles, enabling the modification of existing structures for the generation of libraries of compounds and selection of the best drug candidates.

## Figures and Tables

**Table 1:** The acetylcholinesterase inhibition activity of compounds **1**–**5**.

Compound	IC_50_, μg/mL
**1**	14.58 ± 0.45
**2**	97.67 ± 2.13
**3**	231.94 ± 4.09
**4**	59.69 ± 1.18
**5**	52.06 ± 0.97
Donepezil	2.88 ± 0.16

**Table 2:** The anti-inflammatory activity of compounds **6**–**8** in rats.

Compound	Edema Inhibition, %		
	1 h	2 h	3 h
**6**	14.5 ± 2.4	16.7 ± 1.8	22.1 ± 1.4
**7**	41.9 ± 1.5	50.4 ± 2.1	61.4 ± 1.8
**8**	30.3 ± 1.6	39.6 ± 1.2	45.3 ± 1.1
Indomethacin	45.6 ± 1.7	52.4 ± 2.1	63.2 ± 1.8

**Table 3:** The cytotoxicity of compound **7** on rats.

Compound	LD_50_ (i.p.), mg/kg
**7**	155
Indomethacin	50

**Table 4:** The anti-inflammatory activity of compound **9** in rats.

Compound	Paw Oedema Volume Mean + S.D. (mL)	Percentage Inhibition of Paw Oedema	Dose (mg kg^−1^ p.o.)
2% Gum acacia (control)	0.62 ± 0.029	–	10 mL kg^−1^
**9**	0.46 ± 0.019	25.80	50
Indomethacin	0.25 ± 0.012	59.67	1.5

**Table 5:** The analgesic activity of compounds 6–8 in mice.

Compound	Relative Potency		
	10 min	30 min	60 min
**6**	0.68 ± 0.03	0.73 ± 0.02	0.81 ± 0.04
**7**	0.22 ± 0.01	0.24 ± 0.01	0.27 ± 0.02
**8**	0.85 ± 0.01	0.93 ± 0.03	0.99 ± 0.01
Valdecoxib	1.00	1.00	1.00

**Table 6:** The antidiabetic inhibitory activity of compounds **10**–**14**.

Compound	Antidiabetic Activity (IC_50_ in μM)	
	*α*-Glucosidase	*α*-Amylase
**10**	595.04 ± 43.42	260.40 ± 0.27
**11**	503.43 ± 35.11	121.15 ± 0.65
**12**	216.22 ± 17.32	764.56 ± 0.10
**13**	433.32 ± 88.31	219.89 ± 0.08
**14**	300.45 ± 47.87	109.43 ± 6.12
Acarbose	309.11 ± 22.32	618.87 ± 0.76

**Table 7:** The antibacterial activity of compounds **16**–**18** against Gram-positive and Gram-negative bacteria (inhibition zone diameter, mm).

Compound	*B. cereus*	*S. albus*	*P. aeruginosa*	*E. coli*
**16**	4	3	2	2
**17**	2	4	3	3
**18**	2	2	2	2

**Table 8:** The cytotoxicity of compounds **16**–**18** against *Artemia salina*.

Compound	*Artemia salina* ^a^
**16**	D
**17**	C
**18**	D

^a^ *Artemia salina* (brine shrimp) test. A, high toxicity, 75–100% dead larvae; B, moderate toxicity, 50–75% dead larvae; C, low toxicity, 25–50% dead larvae; D, non-toxic, less than 25% dead larvae.

**Table 9:** The antibacterial activity of compounds **19**–**21** against *Salmonella typhimurium* TA100 ^a^.

Compound	Dose (μg/Plate)											Obs.
	5000	1250	313	78	20	10	5	1.25	0.5	0.31	0.2	
**H-1**	–	–	–	–	–	–	–	K	K	K	K	control
**19**	K	K	K	K	K	K	○	○	–	–	–	
**20**	K	K	K	K	○	○	○	–	–	–	–	
**21**	K	K	K	K	K	–	○	○	○	–	–	

^a^ (K) indicates that *Salmonella* has been killed, (–) indicates that it has not been measured, and (○) indicates that no increase in the number of revertant colonies was observed.

**Table 10:** The antibacterial activity of compounds **19**–**21** against *Salmonella typhimurium* TA98.

Compound	Dose (μg/Plate)											Obs.
	5000	1250	313	78	20	10	5	1.25	0.5	0.31	0.2	
**H-1**	–	–	–	–	–	–	–	K	K	–	●	control
**19**	K	K	K	K	○	K	○	○	–	–	–	
**20**	K	K	K	○	○	–	–	–	–	–	–	
**21**	K	K	K	○	○	–	○	○	–	–	–	

(K) indicates that *Salmonella* has been killed, (–) indicates that it has not been measured, (●) indicates that the number of revertant colonies was more than twice the number of spontaneous revertant colonies in the solvent control group, and (○) indicates that no increase in the number of revertant colonies was observed.

**Table 11:** The biological activities of compounds **22**–**24** against bacteria at 5 mg/mL concentration (inhibition zone diameter, cm) ^a^.

Compound	*S. aureus*	*P. aeruginosa*	*B. subtilis*	*E. coli*
**22**	+	++	-	-
**23**	+	++	-	++
**24**	+	-	-	++
Chloramphenicol	1.0	2.8	2.6	1.0

^a^ Inhibition zone diameter beyond control/(sign): 1.1–1.5 cm/(+++); 0.6–1.0 cm/(++); 0.1–0.5 cm/(+); 0 cm/(-).

**Table 12:** The antimicrobial activity of compound **25** at 100 μg/mL concentration (inhibition zone diameter, mm) ^a^.

Compound	*S. aureus* (MTCCB 737)	*S. epidermidis* (MTCCB 1824)	*E. coli* (MTCCB 1652)
**25**	27	27	26
Tetracycline	30	25	28

^a^ 12 mm or less: resistant or no inhibition, 13–17 mm: moderate inhibition, 18 mm or more: maximum inhibition.

**Table 13:** The minimum inhibitory concentration (MIC, μg/mL) of compound **25** against *Staphylococcus aureus* and *Escherichia coli*, respectively.

Compound	*S. aureus* (MTCCB 737)	*E. coli* (MTCCB 1652)
**25**	50	25
Tetracycline	6.25	12.5

**Table 14:** The cytotoxicity of compound **25** against *Artemia salina*.

Compound	95% Confidence Limit ppm			Regression Equation	X^2^ (df)
	LC_50_	Lower	Upper		
**25**	3.54	2.08	6.02	*y* = 3.98 + 1.85*x*	3.38 (2)
Bleomycin	0.41	0.27	0.62	*y* = 3.16 + 2.98*x*	0.62 (2)
Gallic acid	4.53	3.33	6.15	*y* = 3.93 + 1.62*x*	1.25 (2)

**Table 15:** The antimicrobial activity of compounds **6** and **8** at 100 μg/mL concentration (inhibition zone diameter, mm) ^a^.

Compound	*S. aureus*	*S. epidermidis*	*E. coli*
**6**	10	9	5
**8**	25	23	20
Tetracycline	30	25	28

^a^ 15 mm or less: resistant or no inhibition, 16–20 mm: moderate inhibition, 20 mm or more: maximum inhibition.

**Table 16:** The biological activities of compound **26** against bacteria (minimum inhibitory concentration (MIC), μg/mL).

Compound	*S. aureus*	*B. subtilis*	*S. typhi*	*E. coli*
**9**	25	25	25	50
**26**	100	250	200	100
Amoxicillin	6.25	6.25	6.25	6.25

**Table 17:** The biological activities of compounds **27**–**32** against Gram-positive and Gram-negative bacteria (inhibition zone diameter, mm µg^−1^ sample).

Compound	*S. pneumoniae*	*B. subtilis*	*P. aeruginosa*	*E. coli*
**27**	NA ^a^	10.2 ± 0.53	NA	NA
**28**	14.6 ± 0.22	NA	NA	NA
**29**	NA	9.3 ± 0.44	NA	NA
**30**	NA	8.2 ± 0.53	NA	NA
**31**	NA	NA	NA	NA
**32**	NA	NA	NA	NA
Ampicillin	23.8 ± 0.2	32.4 ± 0.3	–	–
Gentamicin	–	–	17.3 ± 01	19.9 ± 0.3

^a^ NA—no activity.

**Table 18:** The antibacterial activities of compounds **33**–**47** against Gram-positive and Gram-negative bacteria (inhibition zone diameter, mm/µg sample).

Compound	*S. pneumoniae*	*B. subtilis*	*P. aeruginosa*	*E. coli*
**33**	NA ^a^	NA	NA	NA
**34**	12.9 ± 0.19	15.1 ± 0.44	NA	NA
**35**	14.9 ± 0.22	16.2 ± 0.36	NA	12.3 ± 0.27
**36**	NA	NA	NA	NA
**37**	12.3 ± 0.36	14.1 ± 0.17	NA	NA
**38**	NA	NA	NA	NA
**39**	11.3 ± 0.19	12.2 ± 0.44	NA	NA
**40**	NA	NA	NA	NA
**41**	NA	NA	NA	NA
**42**	NA	NA	NA	NA
**43**	NA	NA	NA	NA
**44**	NA	NA	NA	NA
**45**	12.9 ± 0.12	15.1 ± 0.34	NA	NA
**46**	13.2 ± 0.19	15.7 ± 0.44	NA	NA
**47**	NA	10.8 ± 0.34	NA	NA
Ampicillin	23.8 ± 0.2	32.4 ± 0.3	–	–
Gentamicin	–	–	17.3 ± 0.1	19.9 ± 0.3

^a^ NA—no activity.

**Table 19:** The antibacterial activities of compounds **48**–**50** against *S. aureus* (inhibition zone diameter, mm).

Compound	Inhibition Zone Diameter, mm
**48**	23.70
**49**	13.46
**50**	25.94
Penicillin	22.50

**Table 20:** The antifungal activity of compound **51** against four species of fungi (inhibition zone diameter, mm).

Compound	*A. ochraceus*	*P. chrysogenum*	*A. flavus*	*C. albicans*
**51**	34	36	35	24
Mycostatin	36	40	38	40

**Table 21:** The antifungal activities of compounds **22**–**24** (inhibition zone diameter, cm) ^a^.

Compound	*A. fumigatus*	*P. italicum*	*S. racemosum*	*C. albicans*
**22**	++	+	-	+
**23**	++	+	+	-
**24**	+	-	-	-
Terbinafin	3.0	3.6	3.6	3.0

^a^ Inhibition zone diameter beyond control/(sign): 1.1–1.5 cm/(+++); 0.6–1.0 cm/(++); 0.1–0.5 cm/(+); 0 cm/(-).

**Table 22:** The antifungal activity of compound **25** at 100 μg/mL concentration (inhibition zone diameter, mm) ^a^.

Compound	*A. fumigatus*	*A. niger*	*A. alternata*
**25**	17	12	8
Ketoconazole	18	20	21

^a^ 12 mm or less: resistant or no inhibition, 13–17 mm: moderate inhibition, 18 mm or more: maximum inhibition.

**Table 23:** The minimum inhibitory concentration (MIC, μg/mL) of compound **25** against *Aspergillus niger* and *Aspergillus alternata*, respectively.

Compound	*A. niger*	*A. alternata*
**25**	50	50
Ketoconazole	6.25	6.25

**Table 24:** The antifungal activities of compounds **6** and **8** at 100 μg/mL concentration (inhibition zone diameter, mm).

Compound	*A. fumigatus*	*A. niger*	*A. alternata*
**6**	12	9	10
**8**	21	19	19
Ketoconazole	18	20	21

15 mm or less: resistant or no inhibition, 16–20 mm: moderate inhibition, 20 mm or more: maximum inhibition.

**Table 25:** The biological activities of compound **26** against fungi (minimum inhibitory concentration (MIC), μg/mL).

Compound	*A. niger*	*C. albicans*
**26**	250	250
Ketoconazole	31.25	31.25

**Table 26:** The biological activities of compound **9** against fungi (minimum inhibitory concentration (MIC), μg/mL).

Compound	*A. niger*	*C. albicans*
**9**	62.5	125
Ketoconazole	31.25	31.25

**Table 27:** The in vitro antiviral activity of compound **52**.

Compound	CC_50_ ^a^ Μl/Egg	IC_50_	Therapeutic Index ^b^	Titer of the Virus ^c^	S/P Ratio ^d^
IBDV only	–	–	–	10^7^ log2 EID_50_	–
52	>500	≤9	55.5%	10^6^ log2 EID_50_	0.34
Ribavirin	>300	≤7	42.8%	10^2^ log2 EID_50_	–
Negative control	–	–	–	0 log2 EID_50_	0.01
Vaccinated group only	–	–	–	–	0.34

^a^ Cytotoxicity concentration of fifty. ^b^ TI = CC_50_/IC_50_. ^c^ EID_50_ (egg infective dose fifty) with a mixture of IBDV with each compound virus titer. ^d^ S/P ratio: sample/positive ratio.

**Table 28:** Phenotypic activity of compound **53** against intracellular amastigotes of *T. cruzi* (Tulahuen strain) and MRC-5 cells.

Compound	pIC_50_ ^a^		SI ^b^
	*T. cruzi*	MRC-5	
**53**	4.8	<4.2	>4

^a^ All reported values are within a standard deviation of ±0.2 and the result of at least *n* = 2. ^b^ The selectivity index is calculated by dividing the cytotoxicity (IC_50_) by the *T. cruzi* activity (IC_50_).

**Table 29:** The in vitro cytotoxic activity of compounds **54**–**59**.

Compound	Nonviable Cells (%) Concentration (μg/mL)		
	100	50	25
**54**	60%	55%	45%
**55**	60%	50%	35%
**56**	70%	60%	45%
**57**	90%	85%	80%
**58**	50%	35%	10%
**59**	NA ^a^	NA	NA
Doxorubicin	100%	55%	20%

^a^ NA—no activity.

**Table 30:** The in vitro anti-Hep G2 activity results of compounds **56** and **57**.

Compound	IC_50_, μg/mL
**56**	60.27
**57**	8.12
Doxorubicin	43.60

**Table 31:** The in vitro antitumor activity of compounds **60**–**64** (IC_50_, μmol/L).

Compound	L1210	CHO
**60**	<10	<10
**61**	0.14	2.2
**62**	<10	<10
**63**	<10	<10
**64**	1.2	3.5

**Table 32:** The in vitro antitumor activity of compounds **65**–**69** (IC_50_, μmol/L).

Compound	L1210	HL-60	CHO
**65**	7.3	5.3	7.2
**66**	15.4	12.7	6.7
**67**	8.2	5.2	5.0
**68**	4.7	2.8	4.2
**69**	2.6	1.4	1.0
Ciprofloxacin	>150	>150	>150

**Table 33:** The in vitro antitumor activity of compound **70** (IC_50_, μmol/L).

Compound	Hep G2	WI 38	VERO	MCF-7
**70**	65.6	65.0	69.8	66.8
5-Fluorouracil	8.6	3.2	6.5	2.3

**Table 34:** The in vitro antitumor activity of compounds **27**–**32** and **71**–**73** (IC_50_, μmol/L).

Compound	Hep G2	HCT116
**27**	19.4	38.4
**28**	41.8	21.3
**29**	21.6	39.3
**30**	43.9	37.5
**31**	80.9	65.1
**32**	27.6	24.3
**71**	23.3	21.4
**72**	12.4	20.9
**73**	94.9	82.8
Doxorubicin	0.46	0.42
Imatinib	9.7	18.9
5-Flourouracil	1.2	3.5

**Table 35:** The in vitro antitumor activity of compounds **74**–**78**.

Compound	Inhibition Rate, (%, 10 μg/mL)		
	SGC-7901	KB	HT1080
**74**	90.3	89.9	90.6
**75**	95.9	96.3	95.5
**76**	90.5	91.8	88.9
**77**	88.7	89.5	90.3
**78**	96.0	97.5	96.5
Combretastatin A-4	75.0	74.4	76.4
Doxorubicin	87.6	85.3	88.4

**Table 36:** The in vivo antitumor activity of compounds **75** and **78**.

Compound	Dose mg/kg	Administration Method	Number of Animals		Body Weight g		Tumor Weight x ± SD	Inhibition Rate %
			Initial	Final	Initial	Final		
none	–	–	10	10	20.2	26.9	1.80 ± 0.45	–
**75**	10	i.p.	10	10	20.1	27.2	0.65 ± 0.19	63.9
**75**	5	i.p.	10	10	20.3	26.5	0.62 ± 0.23	65.6
**78**	10	i.p.	10	10	20.5	26.5	0.69 ± 0.25	61.7
**78**	5	i.p.	10	10	20.1	27.0	0.76 ± 0.28	57.8
5-Fluorouracil	50	i.v.	10	10	20.7	23.9	0.57 ± 0.22	68.3

**Table 37:** The in vitro antiproliferative activity of compounds **74**–**78**.

Compound	IC_50_ ± SD, μM		
	SGC-7901	A549	HT1080
**74**	33.5 ± 1.9	29.3 ± 1.2	51.3 ± 2.6
**75**	6.12 ± 0.13	1.21 ± 0.09	6.81 ± 0.21
**76**	26.8 ± 1.1	26.4 ± 2.0	26.5 ± 2.2
**77**	48.3 ± 1.5	58.2 ± 2.9	35.6 ± 3.8
**78**	13.8 ± 1.6	17.4 ± 2.0	17.8 ± 1.5
SMART	0.019 ± 0.008	0.029 ± 0.009	0.028 ± 0.011
ABI	0.81 ± 0.08	0.98 ± 0.11	0.15 ± 0.05

**Table 38:** The in vitro antitumor activity of compounds **33**–**47**.

Compound	IC_50_, μg/mL	
	Hep G2	HCT116
**33**	29	39.7
**34**	>100	>100
**35**	22.2	23.3
**36**	72.5	94.6
**37**	45.5	88.6
**38**	38.8	40.1
**39**	48.1	83.1
**40**	22.7	35.2
**41**	31.3	59.5
**42**	>100	>100
**43**	11.3	12.5
**44**	23.6	38.9
**45**	77.4	>100
**46**	46.3	96.5
**47**	11.9	18.9
Doxorubicin	0.42	0.46
Imatinib	18.9	9.7
5-Flourouracil	4.6	4.3

**Table 39:** The in vitro antitumor activity of compounds **79**–**83**.

Compound	IC_50_ ± SD, μM			
	HCT116	Hep G2	HeLa	MCF-7
**79**	45.51 ± 2.80	38.53 ± 2.62	64.90 ± 3.36	34.33 ± 2.32
**80**	82.68 ± 4.21	72.26 ± 4.00	91.45 ± 4.63	65.27 ± 3.71
**81**	74.28 ± 3.84	68.67 ± 3.74	85.90 ± 4.20	57.26 ± 3.30
**82**	7.71 ± 0.62	10.84 ± 0.90	13.11 ± 1.01	9.29 ± 0.73
**83**	92.72 ± 4.70	84.30 ± 4.32	>100	52.51 ± 2.92
Doxorubicin	5.23 ± 0.33	4.50 ± 0.20	5.57 ± 0.46	4.17 ± 0.20

**Table 40:** The ulcerogenic activity of compound **7** in rats ^a^.

Compound	Dose (mg/kg)		
	10	50	100
Control	0/6	0/6	0/6
**7**	0/6(0)	0/6(0)	0/6(0)
Indomethacin	3/6(1.5 ± 0.2) ^b,c^	5/6(1.9 ± 0.2) ^b,c^	6/6(2.2 ± 0.2) ^b,c^

^a^ Number of rat lesions bigger than 0.5 mm in length per total number of rats. ^b^ Mean ulcer lesions ± SEM (mm) (*n* = 6) in parentheses. ^c^ Significant difference at *p* < 0.05 compared with the control.
